# miRNA-Gene Regulatory Network in Gnotobiotic Mice Stimulated by Dysbiotic Gut Microbiota Transplanted From a Genetically Obese Child

**DOI:** 10.3389/fmicb.2019.01517

**Published:** 2019-07-05

**Authors:** Liman Deng, Ruirui Wang, Hui Li, Chenhong Zhang, Liping Zhao, Menghui Zhang

**Affiliations:** ^1^State Key Laboratory of Microbial Metabolism and Joint International Research Laboratory of Metabolic and Developmental Sciences, School of Life Sciences and Biotechnology, Shanghai Jiao Tong University, Shanghai, China; ^2^Department of Biochemistry and Microbiology, School of Environmental and Biological Sciences, Rutgers New Jersey Institute for Food, Nutrition, and Health, Rutgers University–New Brunswick, New Brunswick, NJ, United States

**Keywords:** gut microbiota, gnotobiotic mice, miRNA, gene expression, regulatory network, inflammation, lipid and glucose metabolism

## Abstract

Gut microbiota (GM) dysbiosis has been considered a pathogenic origin of many chronic diseases. In our previous trial, a shift in GM structure caused by a complex fiber-rich diet was associated with the health improvement of obese Prader-Willi syndrome (PWS) children. The pre- and post-intervention GMs (pre- and post-group, respectively) from one child were then transplanted into gnotobiotic mice, which resulted in significantly different physiological phenotypes, each of which was similar to the phenotype of the corresponding GM donor. This study was designed to investigate the miRNA-gene regulatory networks involved in causing these phenotypic differences. Using the post-group as a reference, we systematically identified and annotated the differentially expressed (DE) miRNAs and genes in the colon and liver of the pre-group in the second and fourth weeks after GM inoculation. Most of the significantly enriched GO terms and KEGG pathways were observed in the liver and were in the second week after GM transplantation. We screened 23 key genes along with their 73 miRNA regulators relevant to the host phenotype changes and constructed a network. The network contained 92 miRNA-gene regulation relationships, 51 of which were positive, and 41 of which were negative. Both the colon and liver had upregulated pro-inflammatory genes, and genes involved in fatty acid oxidation, lipolysis, and plasma cholesterol clearance were downregulated in only the liver. These changes were consistent with lipid and cholesterol accumulation in the host and with a high inflammation level. In addition, the colon showed an impacted glucagon-like peptide 1 (GLP-1) signaling pathway, while the liver displayed decreased insulin receptor signaling pathway activity. These molecular changes were mainly found in the second week, 2 weeks before changes in body fat occurred. This time lag indicated that GM dysbiosis might initially induce cholesterol and lipid metabolism-related miRNA and gene expression disorder and then lead to lipid accumulation and obesity development, which implicates a causative role of GM dysbiosis in obesity development rather than a result of obesity. This study provides fundamental molecular information that elucidates how dysbiotic GM increases host inflammation and disturbs host lipid and glucose metabolism.

## Introduction

There is increasing evidence to suspect the pathogenic role of GM dysbiosis in many diseases, particularly in gastrointestinal and metabolic disorders (Lynch and Pedersen, 2016). It has been recognized that GM can affect host phenotypes by modulating host gene expression. MicroRNAs (also called miRNAs) are very important regulators of gene expression that are active in various biological processes, such as development, immunity, adipocyte differentiation, and lipid metabolism (He and Hannon, 2004; Taganov et al., 2007; Peng et al., 2014). Recently, some studies reported that the modulation of gene expression by GM occurred via prior changes in miRNA expression (Dalmasso et al., 2011; Singh et al., 2012). With the rapidly decreasing cost of high-throughput sequencing, there is growing interest in understanding how miRNAs respond to GM and in turn affect gene expression.

Prader-Willi syndrome is a genetically inherited disease caused by deficiencies in imprinted paternal genes in chromosome region 15q11-q13. Patients with PWS have intense hyperphagia, which results in extreme adiposity, a major cause of death (Butler, 2011). Previously, we significantly improved the health status of 17 PWS children using a GM targeted modulation diet (called the WTP diet, composed of whole grains, traditional Chinese medicine food and prebiotics). Evidence and data analysis suggested that the poor clinical status of the PWS children before the intervention was related to dysbiotic GM. To validate this suspicion, the pre- and post-intervention GMs from an intervened PWS child were transplanted into two randomized groups (pre- and post-group, respectively) of genetically identical germ-free male C57BL/6J mice. As expected, the pre-group had a higher inflammation level and more fat accumulation than the post-group (Zhang et al., 2015; Wang et al., 2018). This animal experiment demonstrated the effect of the disturbance of dysbiotic GM on host health. However, the underlying mechanism remains unclear.

This study focused on the effect of the transplanted dysbiotic GM on miRNA expression and aimed to determine the miRNA–mRNA interactions that link GM and host phenotypes. Using sequencing technology, we first obtained the miRNA expression profiles in the liver and colon samples of gnotobiotic mice taken from the previous fecal microbiota transplantation animal experiment. To elucidate the molecular mechanism behind the differences between the two groups, the previously measured gene expression profiling data of the same samples were integrated into this study. Then, we identified DE miRNAs and their target genes between the two groups in the second and fourth weeks after microbiota inoculation and annotated their biological functions. After that, with emphasis on the phenotypic changes in the host, a regulatory network of DE miRNAs, DE target genes and biological functions relevant to lipid and cholesterol metabolism, glucose homeostasis and inflammation was constructed. Finally, GMs that correlated with the key members in the network were studied to decipher how miRNAs along with their target genes were involved in affecting the phenotype of gnotobiotic mice under the pressure of dysbiotic microbiota.

## Materials and Methods

### Animal Experiment

The animal experiment was previously performed as described (Zhang et al., 2015). Briefly, a PWS obese child had received a 90-day WTP dietary intervention. Patient fecal samples before (on day 0) and after (on day 90) the intervention were collected and preserved at −80°C. For the animal transplantation experiment, the two fecal samples were individually diluted in sterile Ringer working buffer. Then, the clarified supernatants of each sample were mixed with an equal volume of 20% skim milk and transferred into two randomized groups (pre- and post-group) of C57BL/6J germ-free male mice through 2 days of continuous oral gavage. During the experiment, the mice were raised in flexible-film plastic isolators with a regular 12-h light cycle and fed a sterile normal chow diet (D12450B, Research Diets, Inc., New Brunswick, NJ, United States) *ad libitum* in SLAC Inc. (Shanghai, China). Half of the mice in each group were sacrificed in the second week (Pre_2W group, *n* = 4 and Post_2W group, *n* = 4) and the rest in the fourth week (Pre_4W group, *n* = 5 and Post_4W group, *n* = 4) after GM transplantation. Colon and liver tissues were collected for RNA sequencing analysis.

The clinical trial of the PWS children was approved by the Ethics Committee of the School of Life Sciences and Biotechnology, Shanghai Jiao Tong University with No. 2012-016 and registered at Chinese Clinical Trial Registry with No. ChiCTR-ONC-12002646. Written informed consent was obtained from the guardian of the PWS donor. The animal experimental protocols were approved and performed in accordance with relevant guidelines and regulations recommended by the IACUC of Shanghai SLAC Laboratory Animal Co., Ltd. All potential biologically hazardous materials in this study were properly handled according to Chinese biosafety laws and regulations.

### Sequencing of miRNAs and mRNAs

For miRNA sequencing, total RNAs were extracted from tissue samples using a mirVana^TM^ miRNA Isolation Kit (Cat. # AM1560; Austin, TX, United States) according to the manufacturer’s instructions. The concentration and purity of the RNA samples were quantified by a NanoDrop spectrophotometer (NanoDrop Technologies, Inc.), and the RNA quality was assessed by an Agilent Bioanalyzer 2100 (Agilent Technologies, Santa Clara, CA, United States). The A260/A280 ratio of the RNA samples was between 2.14 and 2.20. An Illumina TruSeq Small RNA Sample Kit was used to prepare cDNA libraries according to the TruSeq Small RNA Sample Preparation Guide. The concentration and size distribution of the final cDNA libraries were confirmed by a Qubit^®^ 2.0 Fluorometer and an Agilent Bioanalyzer 2100. The sequencing was performed on an Illumina Genome Analyzer IIx with a single-end 1 × 50 nt multiplex procedure.

mRNA sequencing was performed as previously described (Wang et al., 2018). Briefly, total RNAs were extracted using an RNeasy Mini Kit (Qiagen, Germany) and purified using an RNase-Free DNase Set (Qiagen). The cDNA libraries were prepared following the Illumina TruSeq protocol. The mRNA sequencing was performed on an Illumina HiSeq 2500 with a pair-end 2 × 125 nt or 2 × 100 nt multiplex procedure.

The miRNA and mRNA sequencing reads can be accessed in the NCBI SRA database with accession numbers SRP145202, SRP132325 and SRP144862.

### Bioinformatics and Statistics

The raw miRNA reads were first clipped to remove the 3′-adapter. Then, the reads were trimmed with a threshold Q30 and were filtered to exclude reads with an N base or outside 16–36 nt. The high-quality unique sequences were aligned to the mouse mature miRNA database in miRBase (Release 21)^1^ using Bowtie v1.1.1 (Langmead et al., 2009) with parameters -a –best –strata and -v 2. After non-specifically aligned unique sequences were discarded, the remaining specifically aligned sequences constituted the miRNA expression profile.

The raw mRNA reads were first trimmed with a threshold Q20. Then, reads shorter than 30 nt or with an N base were removed. The remaining data were mapped to the *Mus musculus* reference genome GRCm38 (Genome Reference Consortium mouse build 38)^2^ using HISAT version 0.1.7-beta (Kim et al., 2015). The gene expression profiling was calculated using the counts of aligned reads with the mouse genome annotation GTF file^3^ by HTSeq-count version 0.6.1p1 (Anders et al., 2015).

Differential expression analysis between the pre- and post-group was performed using the DESeq2 package (Love et al., 2014). miRNAs and genes with |log_2_foldchange| ≥ 1 were considered to be DE.

For target gene prediction of the DE miRNAs, three target gene prediction algorithms, TargetScan (Agarwal et al., 2015), miRDB (Wong and Wang, 2015), and DIANA-microT-CDS (Reczko et al., 2012), were used to maximally cover the DE miRNAs. To improve the stringency of the prediction, only the genes predicted by at least two algorithms were considered as putative target genes. To ensure that the target genes were specifically expressed in response to the GM, only the putative target genes that were DE were selected as DE target genes.

The GO and KEGG pathway enrichment analyses and functional annotation of the DE target genes were performed using DAVID 6.8 (Huang da et al., 2009). Biological process GO terms and KEGG pathways with gene counts ≥2 and EASE score (modulated *p*-value) < 0.05 were considered to be significantly enriched.

Spearman’s correlation analysis between miRNAs and their target genes was performed, and the *p*-value was adjusted by the method of Benjamini and Hochberg using R statistical language (R 3.2.2). The regulatory network containing miRNAs, target genes and biological functions was constructed by Cytoscape 3.5.1 (Shannon et al., 2003).

## Results

### DE miRNAs and Genes in the Colon and Liver of the Gnotobiotic Mice Between the Pre- and Post-groups

**FIGURE 1:**
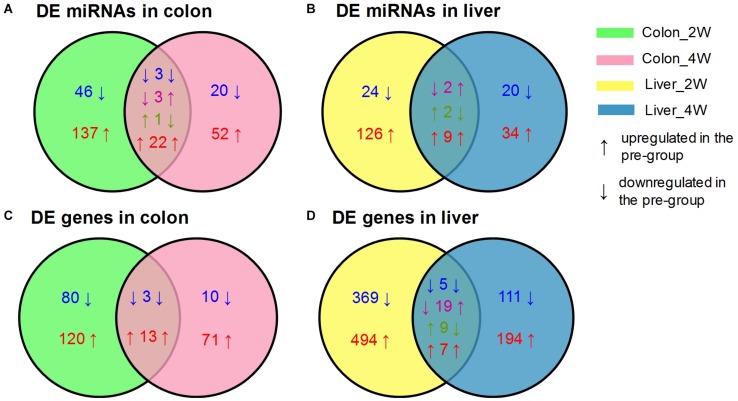
Venn diagrams of the DE miRNAs and genes between the pre-group and the post-group in the second and fourth week after GM transplantation. **(A)** DE miRNAs in colon, **(B)** DE miRNAs in liver, **(C)** DE genes in colon, **(D)** DE genes in liver. DE miRNAs and genes were screened by DESeq2 with the criterion of |log_2_FoldChange| ≥ 1 (pre-group vs. post-group), *n* = 4 or 5 for each group.

After data preprocessing and reference mapping (Supplementary Table S1), we detected 1,818 mouse mature miRNAs and 26,876 genes in colon tissues, whereas 1,831 miRNAs and 21,635 genes were detected in liver tissues. Setting the post-group as a baseline, the pre-group had 313 DE miRNAs and 313 DE genes in the colon and 230 DE miRNAs and 1,248 DE genes in the liver (Table 1). Most of the identified DE miRNAs and genes displayed time- and tissue-specific characteristics in that they appeared only at one time point and in one tissue. Liver tissue had fewer DE miRNAs but many more DE genes than colon tissue, which suggested that the liver might be more affected by the pre-intervention GM. Both colon and liver tissues had many more DE miRNAs and genes in the second week than in the fourth week. Although, we could not obtain the exact influencing time-curve of the GM on host miRNAs and genes due to sampling limitation, this result indicated that the effects of the GM mainly occurred in the initial stage of the transplantation and declined with time. The DE miRNAs identified in colon tissue are an example of this observation. There were 46 downregulated and 137 upregulated miRNAs uniquely identified in the second week, while another 20 downregulated and 52 upregulated miRNAs were identified in the fourth week. In addition, the 29 DE miRNAs that appeared at both time points showed different expression patterns: 3 were downregulated and 22 were upregulated at both time points, 3 were downregulated in the second week but upregulated in the fourth week, and 1 displayed opposite of the previous (Figure 1A). Similar results were also observed for DE miRNAs in the liver (Figure 1B) and for DE genes (Figures 1C,D).

**TABLE 1:** Numbers of DE miRNAs and genes under the stress of the pre-intervention GM.

		**Colon**			**Liver**		
		
		**Total**	**Down**	**Up**	**Total**	**Down**	**Up**
miRNA	2W	212	52	160	163	26	137
	4W	101	24	77	67	22	45
Gene	2W	216	83	133	903	393	510
	4W	97	13	84	345	125	220

**N* = 4 or 5 for each group.*

### DE Target Genes Regulated by the DE miRNAs

Based on the DE miRNA results, large numbers of putative target genes were found. In colon tissue, the 207 DE miRNAs in the second week had 13,606 putative target genes, and the 98 DE miRNAs in the fourth week had 10,855 putative target genes. In liver tissue, 13,352 putative target genes were identified for 161 DE miRNAs in the second week, and 9,923 putative target genes were found for 67 DE miRNAs in the fourth week. Seven DE miRNAs (mmu-miR-8109, mmu-miR-7662-3p, mmu-miR-6539, mmu-miR-5124b, mmu-miR-6402, mmu-miR-142a-3p, and mmu-miR-6352) were removed from further analysis since they did not fulfill our target gene prediction criterion mentioned in the “Materials and Methods” Section.

To be more confident in the target gene selection, the putative target genes were further filtered so that only the DE genes were selected as DE target genes. As a result, in colon tissue, there were 102 DE target genes for 136 DE miRNAs in the second week and 26 DE target genes for 45 DE miRNAs in the fourth week. In liver tissue, 437 DE target genes remained for 148 DE miRNAs in the second week, and 116 DE target genes remained for 53 DE miRNAs in the fourth week (Supplementary Table S2).

### Significantly Enriched Biological Process GO Terms and KEGG Pathways of the DE Target Genes

**FIGURE 2:**
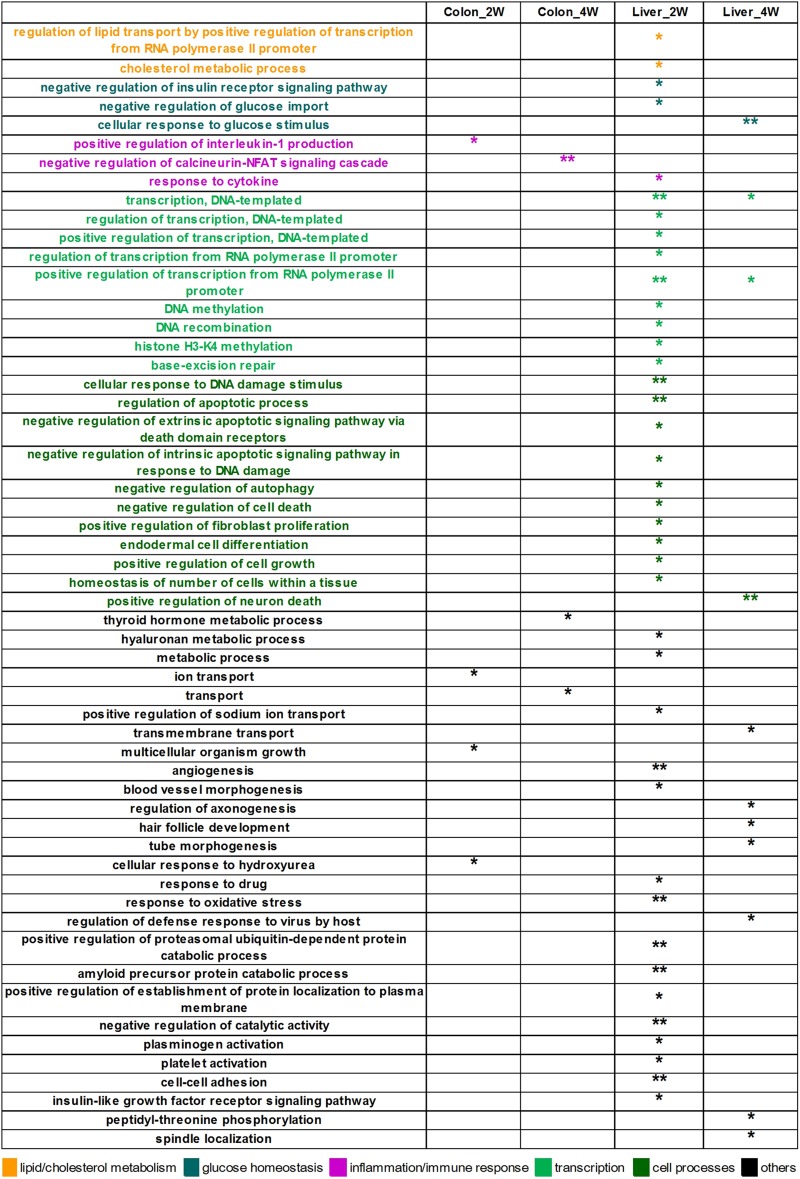
The 55 significantly enriched biological process GO terms of the DE target genes. Most GO terms were identified in the liver, especially in Liver_2W. Some of these terms were associated with lipid and cholesterol metabolism, glucose homeostasis and inflammation. ^∗∗^EASE score < 0.01 and ^*^EASE score < 0.05.

In the analysis of the DE target genes, 55 biological process GO terms were significantly enriched (Figure 2). Most of these terms were identified in liver tissue, especially in the second week (Liver_2W), where 39 of the 55 significantly enriched GO terms were found. Among these significantly enriched terms, the terms “regulation of lipid transport by positive regulation of transcription from RNA polymerase II promoter” and “cholesterol metabolic process” are associated with lipid and cholesterol metabolism. The terms “negative regulation of insulin receptor signaling pathway” and “negative regulation of glucose import” are relevant to glucose homeostasis, and “response to cytokine” is related to inflammation and immune response. In Liver_4W, the number of significantly enriched GO terms was reduced to 11, with one of the terms, “cellular response to glucose stimulus,” being related to glucose homeostasis. Colon tissue had many fewer significantly enriched GO terms, with only four in Colon_2W and three in Colon_4W. Two of these terms, “positive regulation of interleukin 1 production” enriched in Colon_2W and “negative regulation of calcineurin-NFAT signaling cascade” in Colon_4W, are both associated with inflammation and immune response.

**FIGURE 3:**
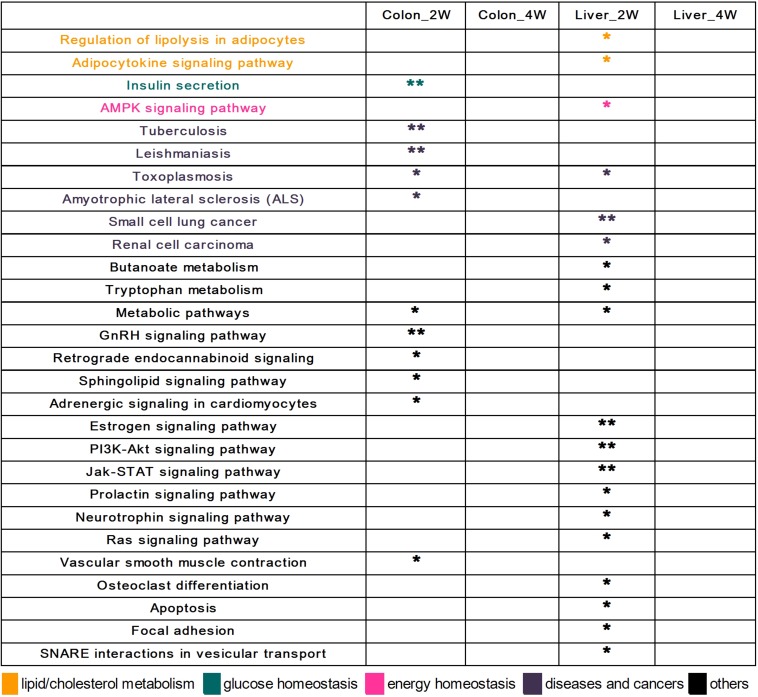
The 28 significantly enriched KEGG pathways of the DE target genes. All pathways were found in the second week in the colon and liver. Some of these pathways were associated with lipid metabolism, glucose homeostasis, and energy homeostasis. ^∗∗^EASE score < 0.01 and ^*^EASE score < 0.05.

A total of 28 KEGG pathways were significantly enriched in the analysis of the DE target genes (Figure 3). Different from the results obtained by GO enrichment analysis, all significantly enriched KEGG pathways were found in the second week. There were 11 KEGG pathways significantly enriched in Colon_2W, and one of them, “Insulin secretion,” is related to glucose homeostasis. In Liver_2W, 19 KEGG pathways were significantly enriched, more than what was observed in Colon_2W. Among these pathways, “Regulation of lipolysis in adipocytes” and “Adipocytokine signaling pathway” are associated with lipid metabolism, and the “AMPK signaling pathway” is crucial in the regulation of energy homeostasis.

### Regulatory Network Among miRNAs, Target Genes, and Biological Functions Relevant to Host Lipid and Cholesterol Metabolism, Glucose Homeostasis, and Inflammation

**FIGURE 4:**
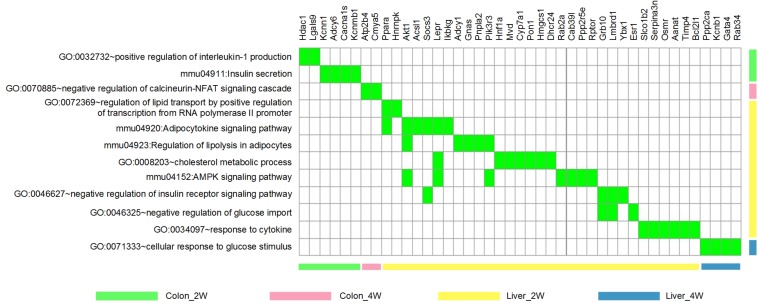
The 12 phenotype-related GO terms and KEGG pathways and the 43 DE target genes involved. These GO terms and KEGG pathways were associated with lipid/cholesterol metabolism, glucose homeostasis, inflammation/immune response and energy homeostasis. Green indicates that the gene is involved in the corresponding term or pathway.

To focus our study on functions relevant to changes in host phenotypes, 12 significantly enriched biological process GO terms and KEGG pathways (associated with lipid metabolism, cholesterol metabolism, glucose homeostasis, inflammation and immune response, and energy homeostasis mentioned in the above section) were selected for further analysis. There were 43 DE target genes involved in these terms and pathways, particularly, 7 of which found in Liver_2W, *Ppara, Akt1, Socs3, Lepr, Pik3r3*, *Grb10*, and *Lmbrd1*, were shared by several terms and pathways (Figure 4).

**FIGURE 5:**
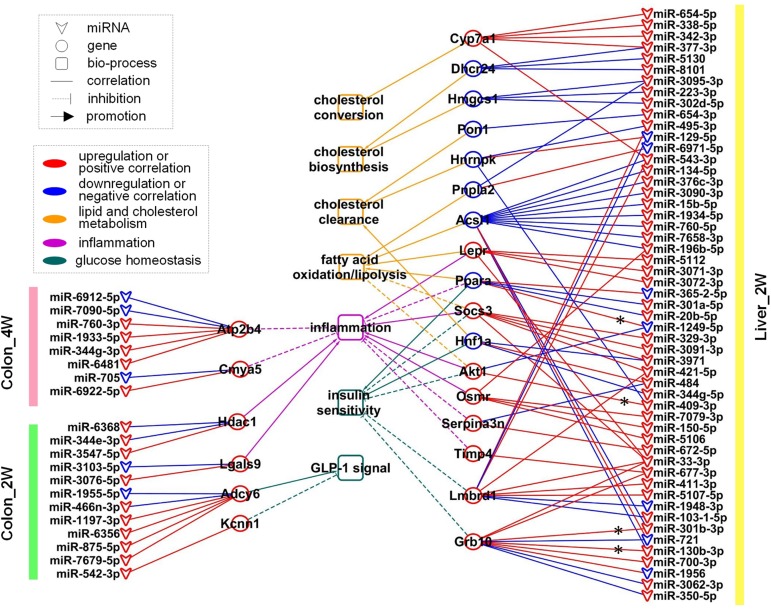
The regulatory network of 73 miRNA regulators, 23 key genes, and 7 biological functions. These miRNAs and genes were mainly related to lipid and cholesterol metabolism, glucose homeostasis and inflammation. ^*^ indicates that the regulatory relationship between miRNA and gene was experimentally validated.

After careful manual checking of the information from functional annotation and literature research (Table 2), 23 out of 43 DE target genes that may be more involved in the phenotype development were finally retained. These 23 key genes were regulated by 73 DE miRNAs (Table 3). A regulatory network of the miRNAs, target genes and biological functions was then constructed as shown in Figure 5 that contained 92 miRNA-target gene regulatory relationships: 51 were positively correlated, while 41 were negatively correlated (Supplementary Table S3). Four of the 92 relationships, miR-1249-5p—*Ppara*, miR-409-3p—*Akt1*, miR-301b-3p—*Grb10* and miR-130b-3p—*Grb10*, had confirmed records in miRTarBase (Chou et al., 2018), an experimentally validated miRNA target gene database. From the constructed network, we found that the relationship between miRNA and mRNA was not simply one-to-one. Many key genes were often regulated by multiple miRNAs. Some of the key genes were even co-regulated by miRNAs with positive and negative correlations. For instance, in Colon_4W, gene *Cmya5* was negatively regulated by miR-705 and positively regulated by miR-6922-5p. Furthermore, a biological function was always contributed by multiple genes, and these genes might also have contradictory relationships with the same function.

**TABLE 2:** Annotations of the 23 key genes associated with lipid and cholesterol metabolism, glucose homeostasis and inflammation.

**Group**	**Gene symbol**	**Gene ID**	**Log_2_FC (pre/post-group)**	**Annotations**	**References**
Colon_2W	Kcnn1	ENSMUSG00000002908	1.02	Insulin secretion, potassium ion transport	Encodes potassium voltage-gated channel and some medicines promote GLP-1 secretion via inhibition of its expression (Shin et al., 2012, 2014).
	Adcy6	ENSMUSG00000022994	1.42	Insulin secretion, inflammatory mediator regulation of TRP channels, regulation of lipolysis in adipocytes	Involved in GLP-1 mediated signaling pathway (Dillon et al., 2005; Doyle and Egan, 2007).
	Hdac1	ENSMUSG00000028800	1.23	Positive regulation of interleukin-1 production, positive regulation of tumor necrosis factor production, negative regulation of insulin secretion	Promote B cell proliferation (Yamaguchi et al., 2010) and IFN mediated innate immunity (Xu et al., 2009).
	Lgals9	ENSMUSG00000001123	1.21	Positive regulation of interleukin-1 production, positive regulation of tumor necrosis factor production, response to lipopolysaccharide, positive regulation of defense response to bacterium, positive regulation of innate immune response, positive regulation of macrophage activation, positive regulation of T cell migration	Encode galectin-9 which could induce T helper cells to secrete pro-inflammatory cytokine IFNγ and TNFα (Su et al., 2011) and synergized with LPS to activate transcriptional factor NF-IL6, then transactivated inflammatory cytokine genes *IL1A*, *IL1B*, and *IFNγ* (Matsuura et al., 2009).
Colon_4W	Cmya5	ENSMUSG00000047419	1.01	Negative regulation of calcineurin-NFAT signaling cascade	Inhibit the activity of NFAT (Kielbasa et al., 2011) which are key regulators of T cell development and function (Macian, 2005).
	Atp2b4	ENSMUSG00000026463	1.07	Negative regulation of calcineurin-NFAT signaling cascade	Inhibit the activity of NFAT (Buch et al., 2005).
Liver_2W	Pon1	ENSMUSG00000002588	–1.36	Cholesterol metabolic process, aromatic compound catabolic process, response to fatty acid	Associated with HDL level and important in lipid and cholesterol metabolism and cardiovascular health (Macharia et al., 2012).
	Dhcr24	ENSMUSG00000034926	–1.29	Cholesterol metabolic process, lipid metabolic process, cholesterol biosynthetic process	Encode enzyme catalyzing the first step of cholesterol biosynthesis (Waterham et al., 2001).
	Hmgcs1	ENSMUSG00000093930	–1.19	Cholesterol metabolic process, lipid metabolic process, cholesterol biosynthetic process	Encode enzyme catalyzing the last step of cholesterol biosynthesis (Mathews et al., 2014).
	Hnf1a	ENSMUSG00000029556	–1.28	Cholesterol metabolic process, reverse cholesterol transport, fatty acid biosynthetic process, fatty acid transport, regulation of insulin secretion, glucose homeostasis, maturity onset diabetes of the young	Encode HNF-1α which activates the transcription of apoM, a major component of HDL particles (Richter et al., 2003), and it can affect plasma levels of HDL, reverse cholesterol transport and cholesterol metabolism (Babaya et al., 2003). Hnf1a^–^/^–^ mice had abnormal HDL particles and suffered from hypercholesterolemia (Shih et al., 2001). Besides, HNF-1α can transactivate insulin I gene (Emens et al., 1992) and it had been reported that Hnf1a knockout mice developed non-insulin-dependent diabetes mellitus (Lee et al., 1998).
	Cyp7a1	ENSMUSG00000028240	1.43	Cholesterol metabolic process, cholesterol homeostasis, cholesterol catabolic process, lipid metabolic process, PPAR signaling pathway, cellular response to glucose stimulus	Encode the rate-limiting enzyme in the conversion of cholesterol to bile acids and plays an important role in bile acid biosynthesis and cholesterol homeostasis (Russell and Setchell, 1992; Pullinger et al., 2002).
	Ppara	ENSMUSG00000022383	–1.22	Regulation of lipid transport by positive regulation of transcription from RNA polymerase II promoter, lipid metabolic process, Adipocytokine signaling pathway, positive regulation of fatty acid oxidation, negative regulation of cholesterol storage, PPAR signaling pathway, non-alcoholic fatty liver disease, glucose metabolic process, response to insulin, positive regulation of gluconeogenesis, regulation of glycolytic process by positive regulation of transcription from RNA polymerase II promoter, insulin resistance, negative regulation of inflammatory response, negative regulation of leukocyte cell–cell adhesion	Play a major regulatory function in lipid catabolism, activation of which can induce fatty acid oxidation, enhance lipolysis and increase energy utilization (Reddy and Hashimoto, 2001; Guzmán et al., 2004). In addition, activation of it can inhibit inflammation and many preclinical experiments demonstrated benefits of PPARα agonists in various inflammation-associated diseases (Gervois and Mansouri, 2012). Moreover, activation of it can lead to improvement of insulin sensitivity (Haluzik and Haluzik, 2006).
	Hnrnpk	ENSMUSG00000021546	–1.20	Regulation of lipid transport by positive regulation of transcription from RNA polymerase II promoter, regulation of low-density lipoprotein particle clearance, cellular response to insulin stimulus	Encode hnRNP K protein, a transactivator of *LDLR* gene which mediates plasma LDL clearance (Li and Liu, 2010).
	Acsl1	ENSMUSG00000018796	–1.14	Adipocytokine signaling pathway, lipid metabolic process, lipid biosynthetic process, fatty acid metabolic process, fatty acid transport, triglyceride metabolic process, PPAR signaling pathway	Activate fatty acids (Ellis et al., 2010a) and directed fatty acid toward β-oxidation (Ellis et al., 2010b). Acsl1 specific knockout could significantly decrease fatty acid oxidation rates (Li et al., 2009; Ellis et al., 2010b).
	Akt1	ENSMUSG00000001729	1.02	Adipocytokine signaling pathway, regulation of lipolysis in adipocytes, negative regulation of fatty acid beta-oxidation, positive regulation of lipid biosynthetic process, non-alcoholic fatty liver disease, glucose metabolic process, glucose homeostasis, insulin resistance, inflammatory response, T cell receptor signaling pathway, B cell receptor signaling pathway, TNF signaling pathway, AMPK signaling pathway	Involved in the development of acute inflammation (Di Lorenzo et al., 2009). Overexpressing a constitutively active *Akt1* in transgenic mice and isolated neonatal cardiac myocytes decreased AMPK activity, resulting in suppressed fatty acid oxidation and glucose uptake and glycolysis (Kovacic et al., 2003). *Akt1* was suspected to decrease insulin sensitivity, since improved glucose tolerance and insulin sensitivity was observed in Akt1^–^/^–^ mice (Buzzi et al., 2010).
	Lepr	ENSMUSG00000057722	2.17	Adipocytokine signaling pathway, cholesterol metabolic process, non-alcoholic fatty liver disease, leptin-mediated signaling pathway, response to leptin, positive regulation of insulin secretion involved in cellular response to glucose stimulus, glucose homeostasis, cytokine–cytokine receptor interaction, T cell differentiation, regulation of energy homeostasis, AMPK signaling pathway	Leptin enhanced immune response via acting on its receptor which is encoded by *Lepr* (Zarkesh-Esfahani et al., 2001) and mice deficient in *Lepr* are resistant to inflammation (Siegmund et al., 2004). Besides, leptin can interact with its receptor LEPR, inhibiting the expression of genes involved in lipogenesis and fatty acid synthesis and increasing the expression of genes mediating fatty acid oxidation, and stimulating fatty acid oxidation by activating AMPK (Liang and Tall, 2001; Minokoshi et al., 2002).
	Socs3	ENSMUSG00000053113	4.18	Adipocytokine signaling pathway, non-alcoholic fatty liver disease, negative regulation of insulin receptor signaling pathway, type II diabetes mellitus, insulin resistance, negative regulation of inflammatory response, TNF signaling pathway	Promote inflammation (Liu et al., 2008; Liu et al., 2015). Negative regulator of leptin receptor signaling, can inhibit fatty acid oxidation and mediate leptin resistance and diet-induced obesity (Bjorbak et al., 2000; Steinberg et al., 2006). Can impair insulin sensitivity via degradation of IRS1 and IRS2 or inhibition of receptor tyrosine phosphorylation (Rui et al., 2002; Senn et al., 2003). Socs3 tissue specific deficiency enhanced insulin sensitivity and protected against obesity- associated insulin resistance (Sachithanandan et al., 2010; Jorgensen et al., 2013).
	Pnpla2	ENSMUSG00000025509	–1.02	Regulation of lipolysis in adipocytes, lipid metabolic process, lipid catabolic process, triglyceride catabolic process, lipid storage, lipid homeostasis	Encode ATGL which catalyzes the first step of triglyceride hydrolysis (Zimmermann et al., 2004). In Pnpla2^–^/^–^ mice, fatty acid release was reduced and massive triglyceride was accumulated (Haemmerle et al., 2006).
	Lmbrd1	ENSMUSG00000073725	1.49	Negative regulation of glucose import, negative regulation of insulin receptor signaling pathway, insulin receptor internalization	Encodes the LMBD1 protein that mediates endocytosis of the insulin receptor. A single-allele knockout of *Lmbrd1* resulted in an enhancement of insulin receptor signaling pathway and increased glucose uptake (Tseng et al., 2013).
	Grb10	ENSMUSG00000020176	1.58	Negative regulation of glucose import, negative regulation of insulin receptor signaling pathway, negative regulation of glycogen biosynthetic process	Interacts with insulin receptor and is a negative regulator of insulin signaling and action (Wick et al., 2003). Loss of Grb10 in mice resulted in enhanced insulin signaling and increased insulin sensitivity (Wang et al., 2007), while overexpression of it caused postnatal insulin resistance (Shiura et al., 2005).
	Serpina3n	ENSMUSG00000021091	1.06	Response to cytokine, acute-phase response	Encode serine protease inhibitor SERPINA3, which inhibits inflammation-associated serine proteases, such as cathepsin G, granzyme B and elastase, to prevent tissue damage during inflammatory responses (Gettins, 2002; Hsu et al., 2014).
	Osmr	ENSMUSG00000022146	1.16	Response to cytokine, cytokine–cytokine receptor interaction	Encode OSM receptor, through which OSM can increase the expression of diverse pro-inflammatory molecules, including IL-6, gp130, and IL1-R1 (Le Goff et al., 2014; West et al., 2017).
	Timp4	ENSMUSG00000030317	1.42	Response to cytokine, response to lipopolysaccharide	Encode matrix metalloproteinases (MMPs) inhibitor, which plays anti-inflammatory function by inhibiting MMPs activity and decreasing TNF-α and IL-1 expression (Celiker et al., 2002).

**TABLE 3:** The 73 miRNA regulators of the 23 key genes.

**Group**	**miRNA**	**Log_2_FC (pre/post-group)**	**Regulated key genes**	**Group**	**miRNA**	**Log_2_FC (pre/post-group)**	**Regulated key genes**
Colon_2W	mmu-miR-3547-5p	2.4738	Hdac1	Liver_2W	mmu-miR-3071-3p	1.72877	Lepr
	mmu-miR-6368	1.22617	Hdac1		mmu-miR-3072-3p	1.20282	Lepr
	mmu-miR-344e-3p	1.04254	Hdac1		mmu-miR-3090-3p	1.26417	Acsl1
	mmu-miR-3103-5p	–1.2423	Lgals9		mmu-miR-3091-3p	1.14713	Socs3
	mmu-miR-3076-5p	1.02201	Lgals9		mmu-miR-3095-3p	1.18135	Hmgcs1, Pnpla2
	mmu-miR-542-3p	2.06234	Kcnn1		mmu-miR-329-3p	1.5288	Socs3
	mmu-miR-7679-5p	1.02946	Adcy6		mmu-miR-33-3p	1.20094	Grb10, Lepr, Lmbrd1, Socs3
	mmu-miR-875-5p	1.21167	Adcy6		mmu-miR-338-5p	1.65017	Cyp7a1
	mmu-miR-6356	1.02966	Adcy6		mmu-miR-342-3p	2.50052	Cyp7a1
	mmu-miR-1197-3p	1.38913	Adcy6		mmu-miR-344g-5p	1.17696	Hnf1a
	mmu-miR-1955-5p	–1.2704	Adcy6		mmu-miR-350-5p	1.16633	Grb10
	mmu-miR-466n-3p	1.20615	Adcy6		mmu-miR-365-2-5p	–1.28404	Ppara
Colon_4W	mmu-miR-6481	1.54255	Atp2b4		mmu-miR-376c-3p	1.65018	Acsl1
	mmu-miR-344g-3p	1.15315	Atp2b4		mmu-miR-377-3p	1.53095	Cyp7a1, Dhcr24
	mmu-miR-1933-5p	1.10568	Atp2b4		mmu-miR-3971	1.11239	Hnf1a, Socs3
	mmu-miR-6912-5p	–1.0309	Atp2b4		mmu-miR-409-3p	1.53844	Akt1, Hnrnpk
	mmu-miR-760-3p	1.43912	Atp2b4		mmu-miR-411-3p	1.75709	Lmbrd1
	mmu-miR-7090-5p	–1.2088	Atp2b4		mmu-miR-421-5p	1.53348	Lmbrd1, Socs3
	mmu-miR-705	–1.0281	Cmya5		mmu-miR-484	1.49636	Hnf1a, Serpina3n
	mmu-miR-6922-5p	1.13032	Cmya5		mmu-miR-495-3p	1.18952	Hnrnpk, Lmbrd1
Liver_2W	mmu-miR-103-1-5p	1.08093	Lmbrd1		mmu-miR-5106	1.99096	Osmr
	mmu-miR-1249-5p	–1.4106	Akt1, Ppara		mmu-miR-5107-5p	1.10158	Lmbrd1
	mmu-miR-129-5p	–1.4207	Hnrnpk, Lmbrd1		mmu-miR-5112	1.97171	Lepr
	mmu-miR-130b-3p	1.05918	Acsl1, Grb10		mmu-miR-5130	1.87501	Dhcr24
	mmu-miR-134-5p	1.41304	Acsl1		mmu-miR-543-3p	1.03476	Acsl1, Cyp7a1, Lmbrd1
	mmu-miR-150-5p	1.84003	Osmr		mmu-miR-654-3p	1.06343	Pon1
	mmu-miR-15b-5p	1.77209	Acsl1		mmu-miR-654-5p	2.19462	Cyp7a1
	mmu-miR-1934-5p	1.28486	Acsl1		mmu-miR-672-5p	1.19394	Serpina3n
	mmu-miR-1948-3p	–1.2602	Lmbrd1		mmu-miR-677-3p	1.13387	Grb10, Timp4
	mmu-miR-1956	–1.0114	Grb10		mmu-miR-6971-5p	–1.02436	Pnpla2
	mmu-miR-196b-5p	1.54465	Acsl1, Osmr		mmu-miR-700-3p	1.07922	Grb10
	mmu-miR-20b-5p	1.17708	Ppara		mmu-miR-7079-3p	1.01557	Osmr
	mmu-miR-223-3p	1.98	Hmgcs1		mmu-miR-721	–1.27276	Acsl1, Grb10
	mmu-miR-301a-5p	1.27932	Ppara		mmu-miR-760-5p	1.37697	Acsl1
	mmu-miR-301b-3p	1.30687	Acsl1, Grb10		mmu-miR-7658-3p	1.45679	Acsl1
	mmu-miR-302d-5p	1.01566	Hmgcs1		mmu-miR-8101	1.47626	Dhcr24
	mmu-miR-3062-3p	1.07836	Grb10				

In Colon_2W, 10 upregulated and 2 downregulated miRNAs jointly regulated 4 target genes, *Adcy6*, *Kcnn1*, *Hdac1*, and *Lgals9* (Figure 5). *Adcy6* has been reported to be involved in the GLP-1 (an incretin secreted by the intestine to promote insulin secretion)-mediated signaling pathway. *Kcnn1* could inhibit GLP-1 secretion. *Adcy6* and *Kcnn1* were both upregulated in the pre-group, indicating disturbed glucose homeostasis in the colon under the stress of the pre-intervention GM. Expression of *Hdac1* and *Lgals9*, which can enhance the immune response and inflammation, was promoted in the pre-group, indicating that the pre-intervention GM increased pro-inflammatory gene expression in the colon in the second week.

In Colon_4W, five promoted and three inhibited miRNAs collectively increased the expression of two anti-inflammatory genes, *Atp2b4* and *Cmya5* (Figure 5), which suggested that inflammation was suppressed in the colon in the fourth week, contrary to that in Colon_2W.

In Liver_2W, 53 microbiota-responsive miRNAs (7 were downregulated and 46 were upregulated) regulated the expression of 17 target genes (eight were downregulated and nine were upregulated) (Figure 5). Among these 17 genes, four pro-inflammatory genes, *Lepr*, *Socs3*, *Akt1*, and *Osmr*, were upregulated, and an anti-inflammatory gene, *Ppara*, was downregulated in the pre-group, which indicated that the pre-intervention GM enhanced the inflammatory response in the liver in the second week. However, opposite results were also observed; the expression of two anti-inflammatory genes, *Serpina3n* and *Timp4*, was increased in the pre-group. Expression of *Pnpla1*, *Acsl1* and *Ppara*, which promote fatty acid oxidation or lipolysis, was suppressed, and expression of *Socs3* and *Akt1*, which inhibit fatty acid oxidation, was increased in the pre-group, which suggested that lipid catabolism was restrained in the liver in the second week under the pressure of the pre-intervention GM. *Pon1* and *Hnf1a*, whose expression is closely associated with HDL levels, and *Hnrnpk*, which can promote plasma LDL clearance, were all downregulated in the pre-group. This downregulation may decrease HDL levels and plasma LDL clearance, resulting in inhibition of reverse cholesterol transport and plasma cholesterol clearance in the pre-group. Similar to what was observed in the inflammatory response, reverse changes were also found in lipid and cholesterol metabolism. Expression of *Lepr*, which promotes fatty acid oxidation, was increased in the pre-group. Two cholesterol synthetase genes, *Hmgcs1* and *Dhcr24*, were downregulated, and a cholesterol invertase gene, *Cyp7a1*, was upregulated in the pre-group, which implied that cholesterol biosynthesis was inhibited and cholesterol conversion was promoted in the liver in the second week under the stress of the pre-intervention GM. In addition to inflammation and lipid and cholesterol metabolism, molecular changes related to glucose homeostasis were also found in Liver_2W. Expression of *Socs3*, *Akt1*, *Lmbrd1*, and *Grb10*, negative regulators of the insulin signaling pathway, was increased, and expression of *Hnf1a* and *Ppara*, which can enhance insulin sensitivity, was decreased in the pre-group, which indicated that the pre-intervention GM inhibited the insulin signaling pathway and reduced insulin sensitivity in the liver in the second week. Among these 17 target genes, the suppression of *Ppara* was validated by RT-PCR and Western blot in our previous work (Wang et al., 2018). Functional annotation of these 17 genes suggested that the liver in the second week mainly exhibited disorders in cholesterol and lipid metabolism, glucose homeostasis and inflammation. In comparison with the colon, the liver had a larger regulatory network with not only more miRNAs and genes involved but also more biological functions influenced.

In contrast to Liver_2W, there was no regulatory network formed in Liver_4W, implying that the response of the liver to the pre-intervention GM mainly occurred in the second week.

### Correlation Relationships Among Key miRNAs/Genes, Key OTUs and Some Physiological/Biochemical Parameters

In our previous work, 45 key OTUs were found to characterize the GMs of the pre- and post-groups. The pre-group had more Bacteroidetes (*Bacteroides* and *Parabacteroides*), Firmicutes (*Ruminococcus*) and Proteobacteria (*Bilophila*), while the post-group had more *Bifidobacterium* and *Lactobacillus*. The transplanted GMs inherited the major features of the corresponding donor samples, and there was no significant change over the first 4 weeks after transplantation (Wang et al., 2018).

**FIGURE 6:**
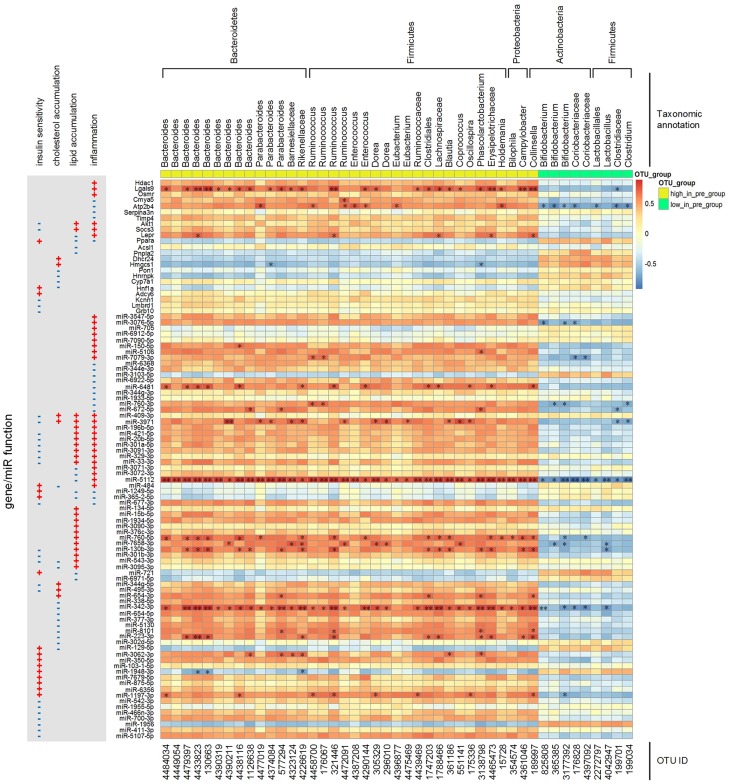
Spearman correlations among the abundances of key OTUs and the expression levels of key miRNAs/genes. The *p*-value was post adjusted by the method of Benjamini and Hochberg. *N* = 4 or 5 for each group. ^∗∗^ adjusted *p*-value < 0.01 and ^*^ adjusted *p*-value < 0.05.

We calculated the correlations among the 45 key OTUs (listed in Supplementary Table S4) and the key miRNAs/genes. As shown in Figure 6, the more abundant OTUs (such as *Bacteroides*) in the pre-group were positively correlated with pro-inflammatory genes (*Hdac1*, *Lgals9*, *Osmr*, *Akt1*, *Socs3*, and *Lepr*), inflammation negative regulator genes (*Cmya5*, *Atp2b4*, *Serpina3n*, and *Timp4*), lipid catabolism-inhibiting genes (*Akt1* and *Socs3*), insulin sensitivity-inhibiting genes (*Akt1*, *Socs3*, *Kcnn1*, *Lmbrd1*, and *Grb10*) and these genes’ positively correlated miRNA regulators. On the other hand, less abundant OTUs (such as *Bifidobacterium*) in the pre-group were positively correlated with lipid catabolism or cholesterol clearance-promoting genes (*Ppara*, *Pnpla2*, *Pon1*, *Hnrnpk*, and *Hnf1a*), insulin sensitivity-enhancing genes (*Ppara* and *Hnf1a*) and these genes’ positively correlated miRNA regulators. Among these key genes and miRNAs, *Lgals9*, miR-5112 and miR-342-3p were significantly correlated with the key OTUs.

**FIGURE 7:**
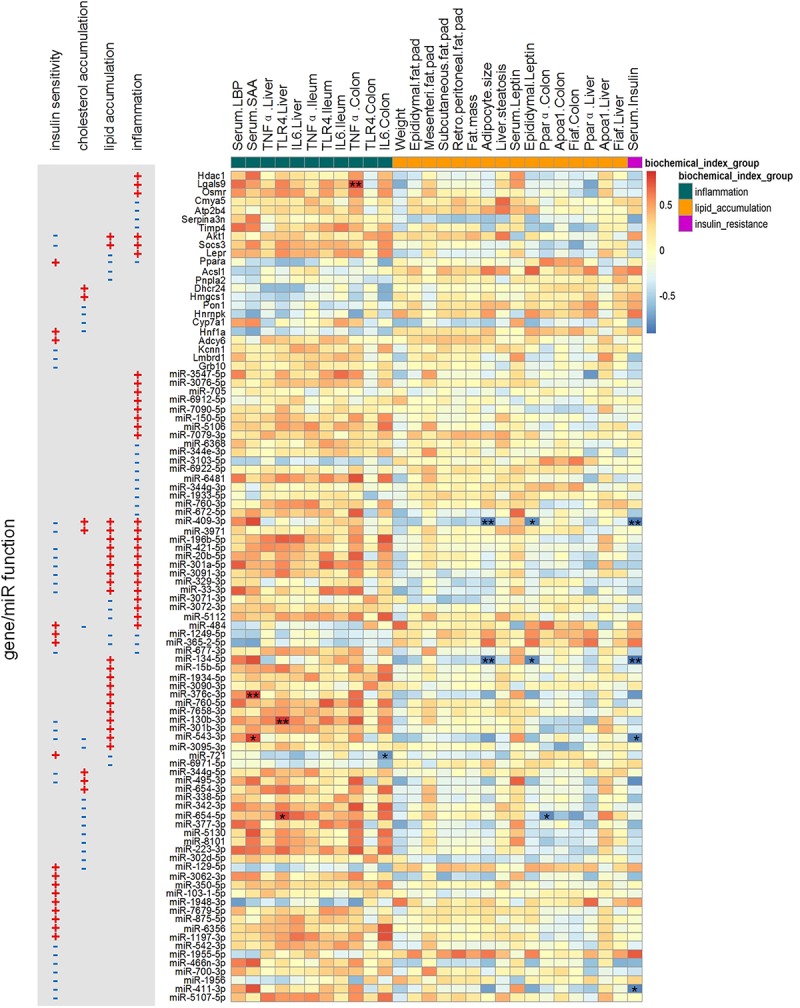
Spearman correlations between physiological/biochemical parameters and the expression levels of key miRNAs/genes. The *p*-value was post adjusted by the method of Benjamini and Hochberg. *N* = 4 or 5 for each group. ^∗∗^ adjusted *p*-value < 0.01 and ^*^ adjusted *p*-value < 0.05.

We also checked the correlations among some physiological/biochemical parameters (listed in Supplementary Table S5) and the key miRNAs/genes (Figure 7). Compared with the post-group, the pre-group had higher expression of inflammatory factors (TNFα, IL6, and TLR4) in the liver and colon and higher concentrations of serum LBP and SAA in the second week after GM transplantation (Zhang et al., 2015). These inflammation-related biochemical parameters exhibited an obvious positive correlation with key pro-inflammatory genes and miRNAs. In the fourth week after GM transplantation, histological images showed that the pre-group developed liver macrovesicular steatosis and they had higher concentrations of hepatic triglyceride and total cholesterol, larger adipocytes, greater fat mass and higher concentrations of serum triglyceride and leptin (Zhang et al., 2015; Wang et al., 2018). However, genes and miRNAs relevant to cholesterol and lipid metabolism, such as *Akt1*, *Socs3*, *Ppara*, *Acsl1*, *Pnpla2*, *Pon1*, *Hnrnpk*, *Hnf1a* and their miRNA regulators, did not show an obvious and consistent correlation with these body fat-related physiological/biochemical parameters.

## Discussion

By combining miRNA and mRNA sequencing data of the colon and liver, this study found that dysbiotic GM indeed changed the expression of miRNAs and their target genes involved in host lipid and cholesterol metabolism, glucose homeostasis and inflammation, which indicated that host miRNAs and genes were important in mediating the effect of the GM and in turn impacting host phenotypes.

In this study, we first screened the DE miRNAs responding to the dysbiotic GM, then sought their targeted DE genes, and finally tracked the key GM members and the biological functions closely correlated with these miRNAs/genes. This strategy resulted in a clear GM-miRNA-gene-biological function regulatory path under the stress of the dysbiotic GM.

We found that the changed expression of miRNAs and their target genes in response to the dysbiotic GM was tissue specific. The pre-intervention GM triggered inflammation in both the colon and liver, but the affected miRNAs and corresponding target genes in these tissues varied. In Colon_2W, the changed miRNAs upregulated the pro-inflammatory genes *Hdac1* and *Lgals9*. In contrast, in Liver_2W, the changed miRNA upregulated the pro-inflammatory genes *Osmr*, *Akt1*, *Socs3*, and *Lepr* and downregulated the anti-inflammatory gene *Ppara*. The GM of the pre-group had more LPS-producing bacteria, such as Gram-negative bacteria *Bacteroides*, *Parabacteroides*, and *Bilophila* (Supplementary Table S4), leading to a higher level of endotoxin load that was reflected by a higher level of serum LBP (Supplementary Table S5). These LPS-producing bacteria had a positive correlation with pro-inflammatory genes along with their positively correlated miRNA regulators (Figure 6), indicating that the enrichment of these LPS-producing bacteria might be responsible for the increase in the expression of pro-inflammatory genes in the pre-group. It has been reported that *Lgals9* could synergize with LPS to activate inflammatory cytokine gene expression (Matsuura et al., 2009). *Hdac1* (Xu et al., 2009; Yamaguchi et al., 2010), *Osmr* (Le Goff et al., 2014; West et al., 2017), *Akt1* (Di Lorenzo et al., 2009), *Socs3* (Liu et al., 2008; Liu et al., 2015) and *Lepr* (Zarkesh-Esfahani et al., 2001; Siegmund et al., 2004) have also been reported to promote the immune response and inflammation. On the other hand, many preclinical experiments have demonstrated the benefits of PPARα agonists in various inflammation-associated diseases (Gervois and Mansouri, 2012).

**FIGURE 8:**
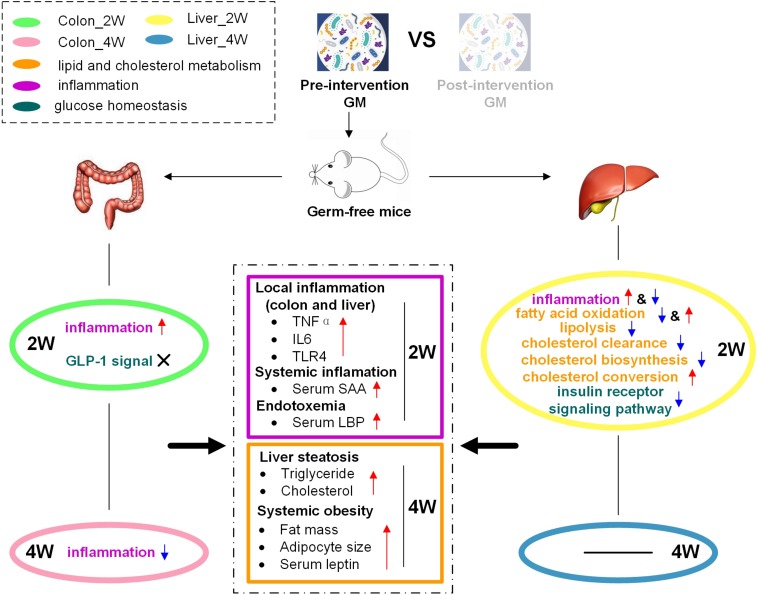
Schematic diagram illustrating how dysbiotic GM affected the host phenotype through the regulation of miRNA and target genes. The pre-intervention GM promoted inflammation and inhibited lipid and cholesterol catabolism and insulin signaling in the colon and liver, resulting in high levels of inflammation and fat accumulation in gnotobiotic mice.

Thus, these inflammation-related molecular changes in the colon and liver might result in higher inflammation levels in the pre-group (Figure 8). Genes and miRNAs that promote inflammation in the colon and liver exhibited obvious positive correlations with inflammatory biochemical parameters (Figure 7). This phenomenon also indicated that the molecular changes induced by dysbiotic GM led to higher levels of inflammation in the pre-group. The host immunity could be regulated in many ways. The intestinal barrier is considered to have an important role in GM-induced host inflammation (Cheru et al., 2019). Lim et al. found that *Lactobacillus sakei* OK67 increased colon tight junction protein expression in high-fat diet-induced obese conventional mice (Lim et al., 2016). In our study, the expression levels of the tight junction proteins ZO-1 and Occludin were very low and were not significantly different between the two groups in the colon, as detected by both mRNA sequencing and RT-PCR (Supplementary Table S6), suggesting that in germ-free mice, the inflammation caused by dysbiotic GM might not, at least not mainly, through the modulation of tight junction protein expression. We could not deduce that the low and unchanged tight junctions expression after fecal microbiota transplantation was mouse specific, as germ-free mice, instead of conventionally raised mice, were used in this study. We were also not sure whether the expression of gut tight junction proteins in germ-free mice was truly lower than that in conventionally raised mice since the latter were not used as a control in our experiment. However, we found that many genes, such as *Hdac1* and *Lgals9*, that can activate inflammatory cytokine gene expression, and *Tlr4*, which is a surface receptor involved in microbial recognition, were expressed at higher levels in the pre-group transplanted with dysbiotic GM. These changes might be the way that the host defended to the GM. Similar to our study, El Aidy et al. (2012) investigated the change in the intestinal mucosa during the conventionalization of germ-free mice and found induced expression of surface receptors, antimicrobial peptides, and pro-inflammatory cytokines and enhanced antigen presentation, although they did not report changes in tight junction proteins.

In addition, the liver had disturbed lipid and cholesterol metabolism, which was not observed in the colon. The expression of target genes that promote fatty acid oxidation or lipolysis, *Ppara*, *Acsl1*, and *Pnpla2*, was suppressed, and the expression of target genes that inhibit fatty acid oxidation, *Socs3* and *Akt1*, was increased in the pre-group in Liver_2W. *Ppara* plays a major regulatory function in lipid catabolism (Reddy and Hashimoto, 2001; Guzmán et al., 2004), and its suppression has also been reported in conventionalized mice that was easier to obtain fat than GF mice (El Aidy et al., 2013). Studies have indicated that specific knockout of *Acsl1* could significantly decrease fatty acid oxidation rates (Li et al., 2009; Ellis et al., 2010b). In *Pnpla2*^–/–^ mice, fatty acid release was reduced, and massive triglycerides accumulated (Haemmerle et al., 2006). *Socs3*, a negative regulator of leptin receptor signaling, can inhibit fatty acid oxidation and mediate leptin resistance and diet-induced obesity (Bjorbak et al., 2000; Steinberg et al., 2006). Overexpression of *Akt1* can decrease AMPK activity, leading to suppressed fatty acid oxidation, glucose uptake, and glycolysis (Kovacic et al., 2003). Thus, these lipid catabolism-relevant molecular changes suggested that dysbiotic GM could promote lipid accumulation in gnotobiotic mouse liver via inhibition of fatty acid oxidation and lipolysis. In addition, we observed that the cholesterol clearance-related genes *Pon1*, *Hnf1a*, and *Hnrnpk* were suppressed in the pre-group in Liver_2W. Many studies have shown that the expression of *Pon1* is closely associated with HDL levels and is important in lipid and cholesterol metabolism (Macharia et al., 2012). Shih et al. (2001) reported that *Hnf1a*^–/–^ mice had abnormal HDL particles and suffered from hypercholesterolemia. *Hnrnpk* has been implicated as a transactivator of the *LDLR* gene that mediates LDL clearance (Li and Liu, 2010). The suppression of these molecules might decrease HDL levels and plasma LDL clearance, resulting in the inhibition of reverse cholesterol transport and plasma cholesterol clearance in the pre-group. These lipid and cholesterol metabolism-related molecular changes might explain why more triglyceride and cholesterol accumulated in the liver or even the whole body in the pre-group (Figure 8). We noted that genes and miRNAs relevant to cholesterol and lipid metabolism did not show an obvious correlation with body fat-related biochemical parameters (Figure 7). Time lag might be the reason for this result as the change of molecular expression occurred in the second week, 2 weeks before changes in body fat. GM dysbiosis might initially induce cholesterol and lipid metabolism-related miRNA and gene expression disorders and then lead to lipid accumulation and obesity development. This lagging obesity phenotype phenomenon implicates a causative role of GM dysbiosis in obesity development, rather than a result of obesity. Considering these observations, we think that a dysbiotic GM from a genetically obese child promoted lipid and cholesterol accumulation in mice on a normal chow diet by inhibiting fatty acid oxidation, lipolysis and plasma cholesterol clearance rather than by enhancing lipogenesis and cholesterol biosynthesis.

Although serum insulin was not significantly different between the two groups of mice (Supplementary Table S5), we identified that GLP-1 and insulin signaling pathways were disturbed in the pre-group. In Colon_2W, two genes relevant to GLP-1 secretion or action, *Adcy6* and *Kcnn1*, were upregulated in the pre-group. In Liver_2W, the expression of four negative regulators of the insulin signaling pathway, *Socs3*, *Lmbrd1*, *Grb10*, and *Akt1*, was increased, and the expression of two genes, *Hnf1a* and *Ppara*, which can enhance insulin sensitivity, was decreased in the pre-group. *Adcy6* has been reported to be involved in the GLP-1-mediated signaling pathway (Dillon et al., 2005; Doyle and Egan, 2007). Some medications promote GLP-1 secretion by inhibiting *Kcnn1* expression (Shin et al., 2012, 2014). It has been reported that *Socs3* can degrade IRSs or inhibit receptor tyrosine phosphorylation (Rui et al., 2002; Senn et al., 2003). *Socs3* tissue-specific deficiency in liver and skeletal muscle could enhance insulin sensitivity and protect against obesity-associated insulin resistance (Sachithanandan et al., 2010; Jorgensen et al., 2013). *Lmbrd1* has been reported to mediate endocytosis of the insulin receptor, and a single-allele knockout of *Lmbrd1* resulted in enhancement of the insulin receptor signaling pathway and increased glucose uptake (Tseng et al., 2013). *Grb10* is a negative regulator of insulin signaling and action (Wick et al., 2003). Overexpression of *Grb10* caused postnatal insulin resistance (Shiura et al., 2005). In *Akt1*^–/–^ mice, improved glucose tolerance and insulin sensitivity were observed (Buzzi et al., 2010). It has been reported that *Hnf1a* could transactivate the insulin I gene (Emens et al., 1992) and that *Hnf1a* knockout mice developed non-insulin-dependent diabetes mellitus (Lee et al., 1998). Many studies have shown that activation of *Ppara* can lead to improvement of insulin sensitivity (Haluzik and Haluzik, 2006). Thus, these molecular changes showed that the pre-intervention GM disturbed GLP-1 signaling and secretion in the colon, inhibited the insulin signaling pathway and reduced insulin sensitivity in the liver.

Moreover, we found that the influence of GM on host molecular expression was time-dependent. Most of the differences between the two groups found in the second week decreased or disappeared in the fourth week, and at different time points, there were different miRNAs and genes responding to the GM. It is well known that diet plays a dominant role in shaping the GM (Zhang et al., 2010). Since the two groups of mice in our study were both on a sterile normal chow diet after microbiota transplantation, there was no environmental pressure to keep the initial GM differences between the two groups. Thus, the GM discrepancy between the two groups decreased as time went on. Another possible reason is that organisms are prone to maintain homeostasis under stimulation via their complex negative feedback regulatory networks. El Aidy et al. found that in addition to the induction of the immune response, the expression of tolerance-associated molecules increased since day 8 after conventionalization. The induction of pro- and anti-inflammatory signals jointly drives a balanced, tolerant immune response to microbiota, establishing immune homeostasis in conventionalized mice (El Aidy et al., 2012). In our study, we also detected increased expression of negative regulators in the pre-group. Two negative regulators of NFAT, *Cmya5* and *Atp2b4*, were upregulated in the pre-group in Colon_4W. The expression of two protease inhibitors, *Serpina3n* and *Timp4*, was increased in the pre-group in Liver_2W. Increased expression of inflammatory negative regulators in the pre-group indicated that inflammatory negative feedback regulation was activated by the higher inflammatory environment induced by the pre-intervention GM. This activation could maintain an organism’s inflammatory homeostasis, prevent tissue damage during inflammatory responses and might explain why most of the inflammatory markers were significantly increased in the pre-group in the second week and were not different between the two groups in the fourth week. Similar negative feedback was also detected in lipid and cholesterol metabolism. *Lepr*, which encodes the leptin receptor was upregulated in the pre-group in Liver_2W. Higher plasma levels of leptin were commonly found in obese humans and rats, and some of these humans and rats even developed leptin resistance (Maffei et al., 1995). Consistently, in our study, *Socs3*, which can mediate leptin resistance, was upregulated in the liver in the second week, and serum leptin levels were significantly higher in the fourth week in the pre-group, which implied that the pre-group might develop leptin resistance. Two genes, *Hmgcs1* and *Dhcr24*, which are involved in cholesterol biosynthesis, decreased, and *Cyp7a1*, which encodes an enzyme that converts cholesterol to bile acids, increased in the pre-group in Liver_2W. These molecular changes might be an organism’s antagonistic responses to cholesterol accumulation. As the mice generated a tolerance to the stimulation by the GM in our study, the GM effects on the host declined as time went on.

Our previous study found that GMs of recipient mice preserved major features of the corresponding donor GMs. Similar to the phenotype differences observed in the PWS donor, gnotobiotic mice that received pre-intervention GM also developed higher inflammation and lipid accumulation (Wang et al., 2018). It is necessary to note that although the PWS child had improved phenotypes after the dietary intervention, it is not certain whether he was already healthy or whether his post-intervention GM was beneficial. However, setting the post-intervention GM as a reference helped us to identify the miRNAs whose expression was disturbed by the dysbiotic pre-intervention GM.

In our animal experiment, fecal microbiota transplantation was performed with only one PWS child as a GM donor. The purpose of this design was to minimize GM variations among individual subjects due to genetic and environmental background (Human Microbiome Project Consortium, 2012) and thus focus on physiological consequences caused by the GM. Indeed, through this pilot investigation, we found that dysbiotic GM could change the expression of miRNAs and their target genes. Bioinformatics analysis indicated that these changes were relevant to host inflammation and to lipid and glucose metabolism. It is worth noting that since this result was from just one donor, the detailed correlation of miRNA and mRNA found in this investigation could be varied upon changing donors. However, the corresponding affected biological functions relevant to these molecules should not change much since they are consistent with the modulation of the host phenotype. In our previous clinical trial, all participants had similar improvement in their physiological metabolic parameters, and their GM structure became similar after the intervention (Zhang et al., 2015). Since the GM donor was randomly selected, we think the results from this donor could be considered representative to a certain degree. Nevertheless, to obtain more generalized conclusions about the relationships among the miRNA, mRNA and host phenotypes, future investigation and validation should be performed with more donors.

In our study, we used deep sequencing rather than microarray to observe host miRNA and gene expression. This approach allowed us to find more DE miRNAs and genes. However, some limitations were also found. Among the large number of miRNAs and genes collected in public databases, information about miRNA function and experimentally verified target genes is extremely scarce. If only verified target genes could be used, many genes obtained by deep sequencing would be out of consideration. Hence, we applied prediction algorithms to identify target genes. However, miRNA target gene prediction algorithms have very high false-positive rates. To improve the stringency, we only considered the target genes predicted by at least two algorithms and further screened them by differential expression. This strategy results in a clear miRNA-gene regulatory path with more confidence; otherwise, the DE miRNAs can cover almost all of the genes detected. To promote miRNA-associated studies, projects that experimentally validate miRNA function and target genes are highly called for in the future.

Through this study, we obtained fundamental molecular information elucidating how a dysbiotic GM increases host inflammation and disturbs host lipid and glucose metabolism by modulating host miRNA and gene expression. These findings provide the possibility for researching microbiota-targeted precision medicine in the future.

## Ethics Statement

The clinical trial of the PWS children was approved by the Ethics Committee of the School of Life Sciences and Biotechnology, Shanghai Jiao Tong University with No. 2012-016 and registered at Chinese Clinical Trial Registry with No. ChiCTR-ONC-12002646. Written informed consent was obtained from the guardian of the PWS donor. The animal experimental protocols were approved and performed in accordance with relevant guidelines and regulations recommended by the Institutional Animal Care and Use Committee (IACUC) of the Shanghai SLAC Laboratory Animal Co., Ltd. All the potential biologically hazardous materials in this study were properly handled according to the Chinese Biosafety Laws and Regulations.

## Author Contributions

RW, CZ, and LZ designed the research. RW performed the experimental procedure. LD, HL, and MZ performed the data analysis and annotation. LD and MZ wrote the manuscript.

## Conflict of Interest Statement

The authors declare that the research was conducted in the absence of any commercial or financial relationships that could be construed as a potential conflict of interest.

## Footnotes

1 ftp://mirbase.org/pub/mirbase/21/mature.fa.gz

2 ftp://ftp.ensembl.org/pub/release-80/fasta/mus_musculus/dna/

3 ftp://ftp.ensembl.org/pub/release-80/gtf/mus_musculus

## Abbreviations

AMPK: adenosine monophosphate activated protein kinase
ATGL: adipose triglyceride lipase
DE: differentially expressed
GLP-1: glucagon-like peptide 1
GM: gut microbiota
HDL: high density lipoprotein
IACUC: Institutional Animal Care and Use Committee
IFN: interferon
IL: interleukin
IRS: insulin receptor substrate
LBP: lipopolysaccharide binding protein
LDL: low density lipoprotein
LPS: lipopolysaccharide
MMPs: matrix metalloproteinases
NFAT: nuclear factor of activated T cells
PWS: Prader-Willi syndrome
SAA: serum amyloid A
TLR: toll-like receptor
TNF: tumor necrosis factor
WTP, whole grains: traditional Chinese medicine food and prebiotics.
